# Modulation of naturalistic maladaptive memories using behavioural and pharmacological reconsolidation-interfering strategies: a systematic review and meta-analysis of clinical and ‘sub-clinical’ studies

**DOI:** 10.1007/s00213-018-4983-8

**Published:** 2018-08-08

**Authors:** Katie H. Walsh, Ravi K. Das, Michael E. Saladin, Sunjeev K. Kamboj

**Affiliations:** 1 Clinical Psychopharmacology Unit, Research Department of Clinical, Educational and Health Psychology, University College London, Gower Street, London, WC1E 6BT UK; 2 Department of Health Sciences and Research, College of Health Professions, Medical University of South Carolina, Charleston, USA

**Keywords:** Memory, Reconsolidation, Plasticity, Anxiety, Phobia, Post-traumatic stress disorder, Substance use disorder

## Abstract

**Background:**

Consolidated memories can undergo enduring modification through retrieval-dependent treatments that modulate reconsolidation. This represents a potentially transformative strategy for weakening or overwriting the maladaptive memories that underlie substance use and anxiety/trauma-related disorders. However, modulation of *naturalistic* maladaptive memories may be limited by ‘boundary conditions’ imposed on the reconsolidation process by the nature of these memories.

**Methods:**

We conducted a systematic review and meta-analyses of behavioural and pharmacological studies examining retrieval-dependent modulation of reward- and threat-related memories in (sub) clinical substance use and anxiety/trauma, respectively.

**Results:**

Of 4938 publications assessed for eligibility, 8 studies of substance use and 10 of anxiety (phobia)- and trauma-related symptoms were included in the meta-analyses. Overall, the findings were in the predicted direction, with most studies favouring the ‘retrieval + treatment’ condition. However, the magnitude of effects was dependent upon the nature of treatment, with pharmacological interventions showing a medium-sized effect (*g* = 0.59, *p* = 0.03) and behavioural treatments, a relatively small effect (*g* = 0.32, *p* = 0.10) in studies of phobia/trauma. Among studies of substance use, post-retrieval behavioural interventions yielded a larger effect (*g* = 0.60, *p* < 0.001) relative to pharmacological treatments (*g* = − 0.03, *p* = 0.91), with treatment type being a statistically significant moderator (*χ*^2^(1) = 4.20, *p* = 0.04).

**Conclusion:**

Modification of naturalistic maladaptive memories during reconsolidation appears to be a viable treatment strategy for substance use and phobias/trauma disorders. However, high levels of heterogeneity and methodological variation limit the strength of conclusions that can be drawn from the reviewed studies at this stage.

## Introduction

### Phobia, traumatic stress and substance use disorders as disorders of memory

Threat-related disorders (phobia and traumatic stress) and substance use disorders (SUDs) can be conceptualised as disorders of maladaptive associative memory (Fanselow and Sterlace 2014; McCarthy et al. 2011; Hyman 2005). The processes underlying the formation and maintenance of these maladaptive memories are thus highly relevant to the treatment of these disorders. The failure of existing therapies to attenuate the emotional/motivational influence of maladaptive memories is one reason why treated individuals are vulnerable to relapse, even after prolonged remission/abstinence (Shaham et al. 2003; Shalev et al. 2002; Staiger and White 1991).

Recent advances in neuroscience have set the stage for the development of a new generation of treatments that focus on reducing the symptom-maintaining influence of maladaptive memories and the attainment of lasting protection against relapse (Kamboj and Das 2017). The current review focuses on a specific retrieval-dependent form of memory plasticity—reconsolidation—that can potentially be manipulated to ameliorate anxiety/trauma and SUD symptoms by targeting the *naturalistic* maladaptive memories that underlie them. The term ‘naturalistic memories’ refers to memories that are naturally (rather than experimentally) acquired. The learning history of naturally acquired maladaptive memories is usually unknown (except perhaps, in the case of recent, single-incident traumas underlying post-traumatic stress disorder, PTSD), although it is assumed that in general, these memories are formed through multiple, intermittent reinforcements (Pavlovian and instrumental) in a variety of contexts and over a prolonged period. This is generally radically different from the situation for experimentally acquired memories, upon which, the vast majority of animal and human reconsolidation research has been conducted.

### Memory reconsolidation

Historically, consolidated memories in long-term storage were thought to be stable and resistant to modification (cf. McGaugh 2000). However, over the past two decades, numerous studies have convincingly demonstrated that under certain retrieval conditions, even apparently long-established, putatively cortically distributed memories can enter a transient labile state during which they are susceptible to modification before being restored in long-term memory (e.g. Graff et al. 2014; Robinson and Franklin 2010; Suzuki et al. 2004). This process is commonly referred to as *reconsolidation* and consists of two temporally and pharmacologically dissociable stages: (a) retrieval-induced *reactivation* or *destabilisation* of a previously consolidated memory and (b) its *restabilisation* in an updated or strengthened form (Lee 2009). Although reactivation engenders a period of memory instability which is required for normative memory strengthening and updating, stored representations are also susceptible to pronounced disruption during reconsolidation using pharmacological agents and behavioural procedures.

### Weakening maladaptive memories: disruption of restabilisation with pharmacological agents

The restabilisation phase of the reconsolidation cycle is protein synthesis-dependent. Drugs that directly and indirectly interfere with protein synthesis can therefore disrupt this phase. The most potent drugs (e.g. anisomycin or cycloheximide) interfere directly with cellular translational machinery and macromolecule biosynthesis. However, these drugs are toxic and not safe for human use. As such, an alternative approach has involved indirect inhibition of protein synthesis through, for example, upstream neurotransmitter blockade. While a number of studies have examined such indirect modulation via diverse drugs (e.g. glucocorticoid, glutamatergic and GABAergic compounds), there are relatively few human studies using these drug classes (cf. Das et al. 2015a, 2018a; Meir Drexler et al. 2015, 2016; Rodríguez et al. 2013; Wood et al. 2015). By contrast, the β-blocker, propranolol, has proven to be a particularly popular tool for probing reconsolidation in humans, especially in laboratory studies of fear conditioning (e.g. Bos et al. 2014; Kindt et al. 2009; Schroyens et al. 2017; Sevenster et al. 2012, 2013, 2014; Soeter and Kindt, 2010, 2011, 2012a, b, 2015a, b). Other studies have extended these experimental findings with propranolol to clinical populations, showing enduring retrieval-dependent reductions in trauma symptoms in people with PTSD (Brunet et al. 2018) and fear in spider phobics (Soeter and Kindt 2015a), as well as drug craving among addicted individuals (e.g. Xue et al. 2017).

### Rewriting maladaptive memories using behavioural techniques

An alternative approach involves disrupting memory expression via reconsolidation interference using purely behavioural strategies (e.g. Monfils et al. 2009). By targeting memory networks that are causally implicated in symptom expression, this approach aims to overcome the limitations of traditional inhibitory training (extinction) strategies. In particular, initially successful extinction is often followed by the ‘return of fear’—or in the case of substance use disorders, the recurrence of craving and drug seeking—following re-exposure to unconditioned stimuli (USs; reinstatement), the simple passage of time (spontaneous recovery) or change in context (renewal). This strongly suggests that maladaptive associative memories persist following typical extinction-based therapies and might contribute to relapse (Bouton 2002; Conklin and Tiffany 2002). Reconsolidation-based behavioural (and pharmacological) treatments can potentially overcome these issues through a *direct updating* of reactivated memory networks.

In support of this idea, extinction learning after fear memory retrieval (so-called ‘retrieval-extinction’) eliminates and prevents the return of fear in rats (e.g. Monfils et al. 2009) and humans (Johnson and Casey 2015; Schiller et al. 2010). Similarly, relative to extinction without prior retrieval, retrieval-extinction leads to enduring reductions in reactivity to drug cues in rodent models of addiction (e.g. Cofresi et al. 2017; Xue et al. 2012) and in human substance users (Germeroth et al. 2017; Xue et al. 2012; see Kredlow et al. 2016 for a recent review of post-retrieval-extinction effects), suggesting that this procedure may be a general-purpose strategy for lasting modification of maladaptive memories. Other therapeutically applicable post-retrieval learning strategies might also be suited to updating appetitive and threat memories in humans, although these have received less attention (cf. Das et al. 2015b; Hon et al. 2016).

### Putative boundary conditions on memory destabilisation

Despite the therapeutic implications of reconsolidation interference hinted at above, there appear to be some inbuilt limits on the regular destabilisation-restabilisation of naturally acquired memories. In particular, ongoing and indiscriminate memory interference following retrieval is constrained by a number of proposed ‘boundary conditions’ that limit destabilisation. Of particular relevance to naturalistic maladaptive memories, older and more strongly encoded associations appear to be relatively resistant to destabilisation following simple retrieval procedures (e.g. Alfei et al. 2015; Milekic and Alberini 2002; Robinson and Franklin 2010; Suzuki et al. 2004). In contrast, experimental studies showing robust reconsolidation effects, particularly in humans, often involve experimentally generated memories (especially conditioned fear), which are often reactivated mere days after training. These simulated maladaptive memories reflect profoundly different learning intensities compared to their naturalistic counterparts in phobia/trauma and SUDs. Associative learning in these disorders involves highly salient USs at encoding (supporting single-trial learning) or reinforcement over many years in multiple contexts. For example, the typical ‘pack-a-day’ smoker will experience close to 10^6^ reinforcements (puffs on a cigarette) over 12 years of regular smoking. These distinct properties of naturalistic memories (asymptotic learning and temporal remoteness) relative to experimentally learned associations (sub-maximal learning and recency) *potentially* severely limit the application of findings from experimental conditioning studies to the treatment of psychological disorders with reconsolidation interference strategies.

In addition, variation in stimulus predictability at retrieval may moderate the ability of retrieval procedures to labilise naturalistic maladaptive memories. In particular, accumulating experimental evidence suggests that a relevant prediction error (PE) at retrieval may be important for enabling full destabilisation of memory networks (e.g. Alfei et al. 2015; Exton-McGuinness et al. 2015; Kindt and van Emmerik 2016; Pedreira et al. 2004; Sevenster et al. 2013, 2014). As an illustration, Das et al. (2015b) found that while simple retrieval cues (followed by counterconditioning) produced intermediate levels of memory updating, incorporation of a PE at retrieval appeared to result in more pronounced rewriting of alcohol memories. More recently, Das et al. (2018a) found no evidence of a reconsolidation-blocking effect of post-retrieval nitrous oxide gas (a putative NMDA receptor antagonist) in heavy drinkers, although reanalysis that took account of the level of *experienced* PE (subjective surprise ratings) at retrieval revealed a significant reduction in craving and drinking behaviour in the retrieval + nitrous oxide group among participants experiencing high PE following reward omission during retrieval. As such, extant studies in humans that demonstrate weakening/updating of naturalistic maladaptive memories without the use of explicit PE-generating procedures during retrieval (the majority of published studies) may reflect a lower bound of efficacy of such interventions, due to sub-optimal reactivation of maladaptive memory networks. However, while evidence of the PE dependence of destabilisation has been demonstrated in studies of experimentally acquired memories in humans (e.g. Sevenster et al. 2013; see also Fernández et al. 2016), this has yet to be tested through systematic variations in the degree of PE during reactivation of memories with fixed and unknown learning histories (i.e. naturalistic memories). More generally, optimal retrieval parameters (e.g. the duration or number of conditioned stimulus (CS) presentations or the use of USs rather than CSs at retrieval; Exton-McGuinness et al. 2015; Merlo et al. 2014) have not been thoroughly studied in humans, leaving some uncertainty about the suitability of the retrieval procedures used in extant studies of naturally acquired memories.

### The current review

To date, reviews and meta-analyses on reward and fear memory reconsolidation have largely focused either on non-human animals (e.g. Das et al. 2013) or in the case of human studies, primarily on experimentally generated memories focusing on a single reconsolidation interference strategy (e.g. Kredlow et al. 2016; Lonergan et al. 2013) or memory system (Scully et al. 2017). Such analyses are critical for furthering our understanding of the modulators of this fundamental memory process. However, a determination of the utility of reconsolidation modulation as a therapeutic strategy requires a synthesis of studies in which clinically important symptoms are targeted in appropriate populations. To our knowledge, no comprehensive synthesis has been conducted on the effects of reconsolidation modulation strategies specifically directed at clinically relevant reward- and threat-related memories in humans. As noted above, the distinct properties of strongly encoded and remote naturalistic maladaptive memories versus those formed during experimental procedures may be extremely important in determining the translational utility of laboratory findings. Moreover, it might be that differences in the neural substrates of learning, and the distinctive learning histories associated with appetitive memories versus threat-related memories, render addictive and phobia/traumatic stress disorders differentially susceptible to reconsolidation treatments due to differences in ‘reactivation potential’ of their underlying maladaptive memories. However, this has yet to be formally tested. Finally, a systematic comparison of behavioural versus pharmacological strategies has yet to be conducted. The current meta-analysis therefore addresses the lack of a systematic synthesis of behavioural versus pharmacological reconsolidation interference strategies applied to human substance-using and anxious/trauma-exposed (clinical and sub-clinical) samples.

## Methods

### Search strategy

PsycINFO, PubMed, Web of Science and Scopus databases were searched on 03/10/2017 using search terms based on a scoping search on experimental and therapeutic modulation of reconsolidation. The search terms were as follows: (memory) AND ((((((((reactivat*) OR destabiliz*) OR destablis*) OR memory reconsolidation) OR reconsolidation) OR reconsolidation-extinction) OR extinction) OR retrieval) AND ((((((((((((((((((pharmacologic*) OR NMDA) OR N-methyl-D-aspartate) OR adrenoceptor) OR adrenergic) OR noradrenergic) OR beta adreno) OR adrenoreceptor) OR sympathetic) OR sympathetic nervous system) OR dopamine) OR dopaminergic) OR glucocorticoid*) OR cortisol) OR benzodiazepine) OR calcium channel) OR extinction) OR exposure) AND (((((((((((avers*) OR appetit*) OR fear) OR anxiety) OR PTSD) OR addiction) OR substance use disorder) OR substance use) OR drug use) OR drug) OR reward).

The search was limited to human studies and excluded reviews. The international clinical trials registry platform and clinicaltrials.gov were searched using the term ‘reconsolidation’, after which a search of the identified authors’ current publications was conducted. The reference lists of the following reviews were also checked for relevant studies: Centonze et al. (2005), de Kleine et al. (2013), de Quervain et al. (2000), Dennis and Perrotti (2015), Farach et al. (2012), Gisquet-Verrier and Riccio (2012), Högberg et al. (2011), Kredlow et al. (2016), Lee et al. (2017), Makkar et al. (2012), Milton (2013), Milton and Everitt (2010), Pitman (2011) and Schwabe et al. (2014). Authors of all included studies were contacted regarding unpublished data.

### Study inclusion criteria

**Fig. 1:**
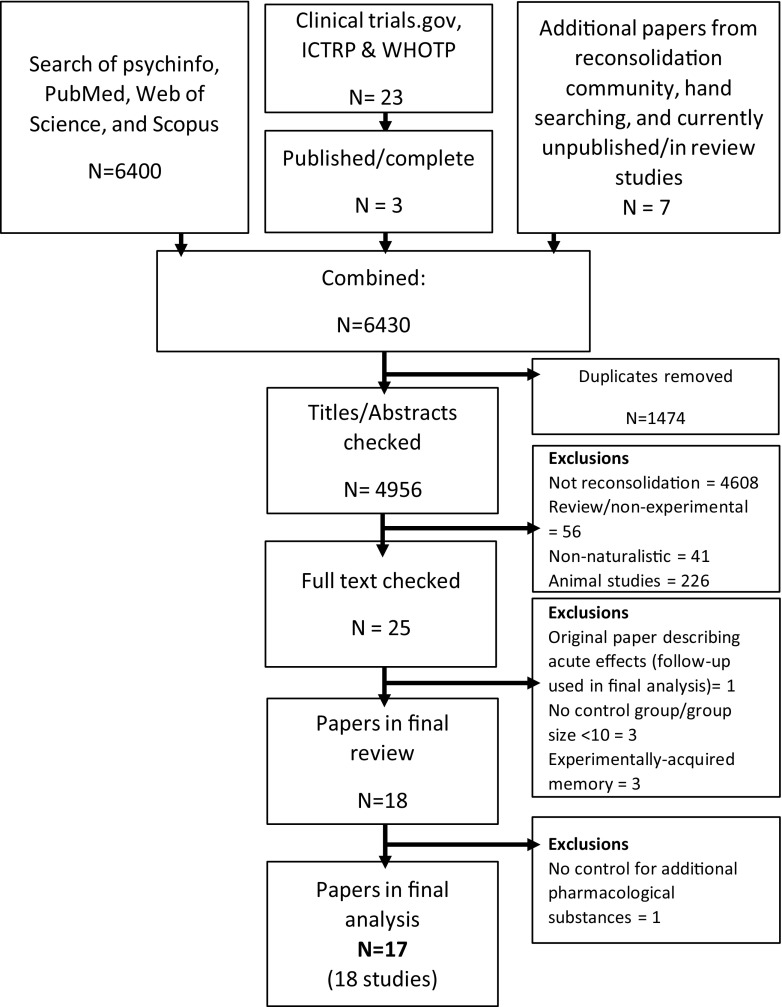
PRISMA flowchart of the study inclusion process

Figure 1 outlines the search, screening and selection process, in line with the Preferred Reporting Items for Systematic Reviews and Meta-Analyses (PRISMA). Selection of studies was restricted to those that examined a reconsolidation-modulating (retrieval-dependent) pharmacological or behavioural strategy targeting naturally, rather than experimentally, acquired memories. In addition, studies were required to assess symptoms relevant to substance use or anxiety/trauma disorders reflecting effects on long-term (≥ 24 h) memory. Participants were required to be recruited on the basis of elevated anxiety, experience of trauma or problematic alcohol/substance use. There was no requirement for a formal diagnosis or for participants to be seeking treatment. Studies were required to randomise adult participants to a ‘retrieval + (reconsolidation-interfering) treatment’ or control group (see below) and contain *n* ≥ 15 per condition *at randomisation* (although the final ES calculation was based on the eventual sample, after exclusions/dropouts). The decision to include only studies with *n* ≥ 15/group was based on pragmatic considerations relating to the limited number of studies with substantial sample sizes. Only studies reported in English were included. Abstracts were reviewed for eligibility by the first author. Eighteen studies that examined pharmacological or behavioural strategies for modifying naturalistic appetitive or threat-related memories via reconsolidation in clinical or sub-clinical human samples were included. Note, one study (Jobes et al. 2015) that initially met inclusion criteria was excluded following discussion due to the complex nature of the design, which involved participants receiving methadone at various times during the intervention (either pre- or post-retrieval). This was in addition to the reconsolidation-interfering study of medication (propranolol), making it impossible to disentangle opioid from β-adrenergic treatment effects. In addition, it should be noted that a recent study on the effects of propranolol on smoking memories (Xue et al. 2017) did not meet criteria because the effects were primarily related to experimentally acquired, rather than naturalistic smoking memories.

### Methodological evaluation of studies

Identifying information (authors, institutions, journal details and mention of significance of results) was obscured from included papers before assessment of the “Methods” section by two (nominally blind) investigators. The tool for methodological appraisal was a modified version of an instrument used in our previous meta-analysis of reconsolidation studies (Das et al. 2013). The level of inter-coder agreement was 83%, and any discrepancies in ratings were resolved through discussion.

### Data extraction

Details regarding the study protocol, including memory retrieval procedure, outcome measures, treatment timing (relative to retrieval), type (behavioural or pharmacological) and dose, as well as ‘disorder’ type were extracted from the selected articles.

### Outcome measures

A preliminary review of the selected studies identified specific outcomes for use in effect size (ES) calculation. These were selected based on the regularity with which these measures were reported across studies. We chose this approach in preference to determining ESs for published significant effects in order to minimise bias, since some of the included studies were not identified as clinical trials, and therefore had no pre-determined (registered) outcomes. As such, subjective craving, an important clinical target in SUD treatment, reflecting conditioned responding to drug cues (i.e. the subjective expression of retrieved drug-related memories) was the primary outcome in the current analysis of substance use studies, as it was reported in all relevant publications. Similarly, studies of phobias consistently used the behavioural approach test (BAT), although the nature of outcomes from this test varied from study to study [e.g. distance between participant and feared object (Shiban et al. 2015); subjective fear ratings during proximal approach (Telch et al. 2017)]. Finally, of the five trauma-related studies, the most commonly reported outcome was PTSD symptom severity (four studies; Table 1) and one study reported memory performance (number of recalled trauma event details; Kredlow and Otto 2015).

**Table 1:** Study details

Study	*N* Cont:Tx	% Male	Mean Age	Retrieval-dependent treatment	Control condition	Reconsolidation procedure	Outcome (ES calculation)	Effects reported in publication (bold=result used for ES calculation)
Phobia/trauma studies								
Björkstrand et al. (2016) Specific (spider) phobia	20:20	26.7%	26.20	Retrieval-extinction	RETRIEVAL+ 6hr + Tx*	Retrieval (12s) ➔ 10-min ➔ Exposure	Approach behaviour (BAT)	↓ in BOLD response to familiar and novel cue (1 day) ↑ in approach behaviour (1 day) **↑ Proportion of spider versus neutral pictures views (6 months)** ↓ in BOLD response to cue in right amygdala (6 months) No ∆ BOLD response to cue in left amygdala (6 months)
Brunet et al. (2018)	30:30	41.7%	39.4	Propranolol	RETRIEVAL+NTx	0.67 mg/kg propranolol (SA) ➔ 120 min; session 1) ➔1.0 mg/kg propranolol (LA; concurrently administered after session 1) ➔ 90 minutes ➔ Retrieval (10-20 min)	Symptom severity (CAPS)	**↓ CAPS**, PCL-S (**6 weeks**; 6 months)
Kredlow & Otto (2015) Indirect witnesses of a terrorist act	22:23	20.2%	19.30	Negative story - interference	RETRIEVAL + NTx	Retrieval (4-min) ➔ Negative story	# details for traumatic event	↓ number of details for event (1 week)
Maples-Keller et al (2017) Specific (flying) phobia	30:27	21.1%	42.11	Retrieval-extinction (VRET)	NRETRIVAL + Tx	Retrieval (15s) ➔ 10-min ➔ VRET	Fear (FFI)	No ∆ FFI, QAF (posttreatment; 3 months; 6 months) **No ∆ FFI**, QAF (12 months) ↑HR (3 month) ↓SCR (3 month)
Shiban et al (2015) Specific (spider) phobia	15:13	10.0%	31.14	Retrieval-extinction (VRET)	NRETRIEVAL +Tx	Retrieval (5s) ➔ 10-min ➔ *In vivo* and VRET	Approach behaviour (BAT)	**No** ∆ **in approach behaviour during BAT (1 day)** No ∆ fear ratings during spontaneous recovery (1 day) No ∆ in SCR (1 day) No ∆ in-vivo fear ratings (1 week) No ∆ self-reported avoidance or FSQ (6 months)
Soeter & Kindt (2015a) Specific (spider) phobia	15:15	9.0%	21.60	Propranolol	NRETRIEVAL +Tx	Retrieval (2-min) ➔ 40mg propranolol	Approach behaviour (BAT)	No ∆ in numerical fear scale (4 days) ↑ in approach behaviour during BAT (11 days) No ∆ in SPQ (11 days) ↑ in approach behaviour during BAT (3 months) No ∆ in self-reported fear (3 months) **↑ in approach behaviour during BAT (1 year)** ↓ in numerical fear scale (1 year)
Surís et al (2013) Patients (Vietnam and post-Vietnam era veterans) with PTSD	24:27	100%	43.02	Rapamycin	RETRIEVAL+NTx	15 mg rapamycin (‘sirolimus’) ➔ retrieval(30-75 min; average:45-min)	Symptom severity (CAPS)	↓ CAPS in post-Vietnam subgroup (1 month) **No ∆ CAPS**, PCL, QIDS-SR (1 month) No ∆ CAPS, PCL, QIDS-SR (3 months)
Telch et al (2017) Specific (spider/snake) phobia	17:15	12.5%	21.31	Retrieval-extinction	Tx+RETIEVAL**	Retrieval (10s) ➔ 30 minutes ➔ In vivo exposure (3-min x6)	Peak fear during behavioural approach (BAT)	No ∆ peak fear during BAT (1 day) No ∆ expected fear during BAT (1 day) **↓ in peak fear during BAT renewal test (1 month)** No ∆ expected fear during BAT (1 month) No ∆ FSQ (1 month)
Wood et al (2015; Study 2) Patients (civilians and veterans) with PTSD	15:13	71.43%	45.75	Mifepristone	NRETRIEVAL+Tx	1800mg mifepristone ➔ 90-min ➔ retrieval (duration not specified)	Symptom severity (IES)	**No ∆ IES-R**, Physiological ‘PTSD probability score’, HR, SCR, F-EMG, C-EMG (1 week)
Wood et al (2015; Study 3)/ Patients (civilians) with PTSD	15:16	45.2%	38.50	D-Cycloserine & Mifepristone	RETRIEVAL+NTx	100 mg D-Cycloserine ➔ 240-min ➔ 1800mg mifepristone ➔ 90-min ➔ retrieval (duration not specified)	Symptom severity (IES)	**No ∆ IES-R**, Physiological ‘PTSD probability score’, HR, SCR, F-EMG, C-EMG (1 week)
Substance Use								
Das et al (2015a) Dependent current smokers	20:19	50.0%	28.39	Memantine	NRETRIEVAL+Tx	10 mg memantine ➔ 210-min➔ retrieval (5-min)	Craving (QSU)	**No ∆ craving (1 week)** No ∆ cue-induced BP; SCR; HRV (1 week ) No ∆ smoking-related attentional bias (1 week) No ∆ relapse latency (3 month) No ∆ nicotine dependence (3 month)
Das et al (2015b) Nondependent current hazardous drinkers	19:20	50.0%	22.33	Counter-conditioning	NRETRIEVAL+Tx	Retrieval (5-min)➔10-min➔ Counterconditioning	Craving (ACQ)	**↓ craving (expectancy; 1 week)** ↓ alcohol attentional bias (1 week) ↓ Cue liking (1 week) No ∆ in self-reported drinking (1 week)
Das et al (2018a)	20:21	66%	27.25	Nitrous Oxide	NRETRIEVAL+Tx	Retrieval (5-min) ➔ 10-min ➔ Nitrous Oxide (30-min)	Craving (ACQ)	**No ∆ craving (10 days)** No ∆ drinking behaviour, cue liking or cue-induced urge to drink (10 days) NB following post-hoc group reassignment based on level of prediction error at retrieval (high and low self-rated surprise during retrieval; 10 days) ↓ craving, drinking behaviour; cue-induced urge to drink in high PE - N_2_O group (10 days) No ∆ cue liking in high PE - N_2_O group (10 days)
Germeroth et al (2017) Dependent current smokers	39:34	64.2%	47.50	Retrieval-extinction	NRETRIEVAL+Tx	Retrieval (5-min)➔10-min➔ Cue exposure	Craving (QSU)	↓ # cigarettes/day (2 weeks) No ∆ craving for novel and familiar cues (2 weeks) No ∆ CO level (2 weeks) **↓ craving for novel and familiar cues (1 month)** ↓ # cigarettes/day (1 month) ↓ expired CO level (1 month) No ∆ cotinine (1 month) No ∆ cue-induced BP; HR (1 month)
Hon et al (2016) Nondependent current hazardous drinkers	16:16	61.7%	27.00	Cognitive reappraisal	NRETRIEVAL+Tx	Retrieval (5-min)➔ 10-min ➔ Reappraisal	Craving (ACQ)	**↓ craving (purposefulness; 1 week)** ↓ verbal fluency for +ve alcohol words (1 week) No ∆ drinking (1 week) No ∆ attentional bias (1 week)
Pachas et al (2015) Dependent current smokers	31:23	73.0%	42.05	Propranolol (0.67mg/kg)	RETRIEVAL+NTx	0.67 mg/kg propranolol (SA) ➔ 90 min➔1.0 mg/kg propranolol (LA)➔ retrieval (duration not specified)	Craving (VAS)	**No ∆ craving (1 week)** No ∆ cue (smoking-script)-induced HR, SCR, EMG (1 week)
Saladin et al (2013) Dependent current cocaine users	24:26	66.0%	39.95	Propranolol (40mg SA)	RETRIEVAL+NTx	Retrieval (20-min)➔ 40 mg (SA) propranolol	Craving (CDMS)	↓ in craving, systolic and diastolic BP (1 day) No ∆ in HR (1 day) **No ∆ cue-induced craving, HR, or BP (1 week)**
Xue et al (2012) Dependent, abstinent heroin users	22:22	100.0%	37.70	Retrieval-extinction	NRETRIEVAL+Tx	Retrieval (5-min) ➔ 10-min ➔ Cue exposure (60 min)	Craving (VAS)	↓ in heroin craving, Diastolic BP, Systolic BP, HR (4 days) No ∆ in HR (4 days) ↓ in heroin craving, Systolic BP (34 days) No ∆ in Diastolic BP (34 days) No ∆ in HR (34 days) **↓ in heroin craving (184 days)** ↓ in Systolic BP (184 days) No ∆ in HR, Diastolic BP (184 days)

Note for Pachas et al 2015 and Wood et al, 2015 retrieval duration details were not provided but based on references to previous script-driven retrieval (Pitman, et al., 1987). Note on control groups: Some studies used a three group design. Only the control group used in the ES calculation is described in the table

*NRetrieval*= no retrieval, *Tx*= treatment, *NTx*=no treatment, *VAS*=Visual analogue scale, *QSU*=Questionnaire on smoking Urges; *ACQ*=Alcohol Craving Questionnaire, *CDSM* = Craving/Distress/Mood States, *BAT* Behavioural Approach Test, *BOLD*, Blood Oxygen Level Dependent (fMRI response), *IES*=Impact of Events Scale, *FFI*= Fear of Flying inventory, *PE*=prediction error *VRET*=Virtual reality exposure therapy; *SA*=short acting; *LA*=long acting, *SCR*=skin conductance response, *F-EMG*=frontalis EMG, *C-EMG*=corrugator EMG

* retrieval followed by a 6 hr delay, followed by treatment

** treatment preceded retrieval

### Statistical approach

#### Effect size determination

Data required for ES determination were extracted by the first author. Random effects models (DerSimonian and Laird 1986) were selected, and the generic inverse variance method was used. ESs were calculated as between-groups standardised mean differences (Hedge’s *g*; Higgins and Green 2011) using the Review Manager software (version 5.3; the Cochrane collaboration, 2014) and interpreted using the standards of Cohen (1988) and Sawilowsky (2009): ~ 0.1 = very small, ~ 0.2 = small, ~ 0.5 = medium, ~ 0.8 = large and ~ 1.2 = very large. Intermediate descriptive labels (e.g. small–medium) were used to describe ESs, where appropriate.

ESs related to the primary 1 *df* comparison of interest, namely retrieval + treatment (pharmacological or behavioural) versus a suitable control condition. A comparison with a no retrieval + treatment control was deemed to best represent the specific effect of a memory-interfering/weakening treatment via reconsolidation. Where such a group was not used, ESs were calculated relative to a retrieval + no treatment condition. Other control groups are also suitable for testing reconsolidation effects. Unlike pharmacological studies, in which drug effects are likely to be present for several hours (i.e. during the period of memory lability) even if the treatment is administered prior to reactivation, retrieval-dependent memory-interfering behavioural treatment effects are theoretically constrained if the treatment occurs before retrieval. As such, treatment *followed by* retrieval is a suitable control condition in behavioural studies (however, see Hutton-Bedbrook and McNally (2013) for discussion of effects that are not consistent with a standard reconsolidation interpretation). Finally, comparison groups in which treatment is delivered after retrieval but outside of the ‘reconsolidation window’ are also suitable controls for retrieval-dependent memory effects, as destabilisation/reactivation is a time-limited process (lasting < 6 h).

Given that reward- and threat-related disorders have distinct multipath aetiologies and underlying learning processes, these disorder types were evaluated separately in meta-analyses. Alternatively, given the aetiological similarity in terms of the proposed central role of classical conditioning in specific phobias and trauma-related disorders, these two classes of disorders were considered together as a single category (phobia/trauma). Further, using sub-group analysis, we examined whether treatment type (behavioural versus pharmacological) produced different population ES estimates within each broad disorder type. Finally, we examined moderation by gender ratio, participant age and score on the methodological appraisal tool (based on the number of positively endorsed desirable study characteristics as a proportion of the total number of items that could be positively endorsed) across *all studies*, using these as continuous variables in meta-regressions. Note, although variation in retrieval parameters (especially retrieval trial duration and time between reactivation and treatment) could affect the extent to which memories are reactivated or weakened/overwritten, insufficient variability in these parameters prevented us from exploring these as moderators (cf. Kredlow et al. 2016).

Sub-group analyses and forest plots were derived from RevMan. Heterogeneity across studies was assessed using the *I*^2^ statistic and described qualitatively; thus, ~ 25% = low, ~ 50% = moderate and ~ 75% = high (Higgins et al. 2003). Sensitivity analysis was conducted when heterogeneity was relatively high and involved testing the effects of sequentially removing individual studies to determine which had the greatest influential on heterogeneity. Alternative aggregate ESs are reported where removal of the most influential study resulted in a reduction of heterogeneity to moderate levels or below (i.e. *I*^2^ < 50%).

Where insufficient information was available in publications to calculate ESs from means/SDs and these details were not available from authors (Pachas et al. 2015; Xue et al. 2012), estimates were obtained from figures in the relevant publications using Plot Digitizer software (Poisot 2011). Publication bias (symmetry of funnel plots and trim and fill) was assessed using the MAVIS package version 1.1.3 (Hamilton et al. 2017).

#### Terminology

‘Reactivation’ refers to the first stage of the reconsolidation process, as well as a memory state that is highly accessible and malleable (Gisquet-Verrier and Riccio 2012). In addition, the term is used to describe procedures intended to achieve this memory state. Since the terms ‘reactivated’ and ‘destabilised’ are both used to describe a labile, potentially modifiable state of long-term memories, we do not distinguish between these terms and use them interchangeably. ‘Retrieval’ is used here to refer to experimental procedures that are intended to reactivate/destabilise memories, but which may or may not be successful in this regard. This term is not intended to imply recall of a discrete memory trace (cf. Telch et al. 2017), but, rather, retrieval or reactivation of a more complete network of reward (substance use) or threat-related (phobic/trauma-related) associations.

## Results

### Study and sample characteristics

After exclusions, the literature search yielded a total of 18 studies from 17 publications (*n* = 774). Five were studies on specific phobias, five on trauma-related symptoms and eight on substance use. Of the phobia/trauma studies, five examined pre- or post-retrieval pharmacological interventions (Brunet et al. 2018; Soeter and Kindt 2015a; Surís et al. 2013; Wood et al. 2015; studies 2 and 3) and five examined post-retrieval behavioural strategies (Bjorkstrand et al. 2017; Kredlow et al. 2016; Maples-Keller et al. 2017; Shiban et al. 2015; Telch et al. 2017). The eight substance use studies also examined either pharmacological (*k* = 4; Das et al. 2015a, 2018a; Pachas et al. 2015; Saladin et al. 2013) or behavioural (*k* = 4; Das et al. 2015b; Germeroth et al. 2017; Hon et al. 2016; Xue et al. 2012) reconsolidation interference strategies.

Participant details (gender ratio; age) are presented in Table 1. There was a considerable variation between studies in terms of gender ratio of participants. Among substance use studies, gender was generally balanced or there was a higher proportion of men, in line with the gender prevalence of SUD in epidemiological studies (Seedat et al. 2009). Xue et al. (2012) was an exception as it only included male participants (detoxified heroin users). In contrast, studies of phobia/trauma were generally skewed towards a higher representation of women, again, in line with epidemiological evidence (McLean et al. 2011). An exception was the study by Surís et al. (2013), which only recruited men (combat veterans). Participant age varied widely across studies, although the mean age of participants was not statistically different (*p* = 0.80) between phobia/trauma studies (*M* = 32.83,  *SD* = 10.12) and substance use studies (*M* = 34.02,  *SD* = 8.93).

### General study methodologies

Key design features of studies and the presence/absence of specific desirable methodological study features are outlined in Tables 1 and 2. Table 2 shows that studies generally contained many desirable methodological features. The most common methodological limitations across studies were a lack of comprehensive experimental conditions that controlled for the effects of simple retrieval or treatment alone. In addition, a lack of experimenter/assessor blinding was a virtually universal limitation of the behavioural studies, but uncommon in pharmacological studies.

**Table 2:** Methodological/reporting features of studies

Study name	A	B	C	D	E	F	G	H	I	J	K	L	M
Brunet et al. (2018)	Y	Y	Y	Y	Y	Y	N	Y	N	Y	Y	Y	Y
Das et al. (2015a)	Y	Y	N	Y	Y	N	Y	Y	N/A	Y	Y	Y	Y
Das et al. (2015b)	Y	Y	Y	N	Y	N	N	Y	N/A	Y	Y	N/A	Y
Das et al. (2018a)	Y	Y	N	N	Y	N	N	Y	N/A	Y	Y	Y	Y
Germeroth et al. (2017)	Y	Y	Y	N	Y	Y	N	Y	N/A	Y	Y	N/A	N
Hon et al. (2016)	Y	Y	N	N	Y	Y	N	Y	Y	Y	N	N/A	Y
Pachas et al. (2015)	Y	Y	N	Y	Y	Y	N	Y	N/A	N	Y	Y	N
Saladin et al. (2013)	Y	Y	Y	Y	Y	Y	N	Y	N/A	Y	Y	Y	N
Xue et al. (2012)	Y	Y	N	N	Y	Y	N	Y	N/A	Y	Y	N/A	Y
Soeter and Kindt (2015a)	Y	Y	N	N	Y	Y	Y	Y	N	Y	Y	Y	N
Telch et al. (2017)	Y	Y	Y	N	Y	Y	Y	Y	N/A	Y	Y	N/A	Y
Bjorkstrand et al. (2017)	Y	N	N	N	Y	N	N	N	N/A	Y	Y	N/A	Y
Shiban et al. (2015)	Y	Y	Y	Y	Y	N	N	Y	N/A	Y	Y	N/A	N
Maples-Keller et al. (2017)	Y	Y	N	Y	Y	Y	N	Y	N	Y	Y	N/A	Y
Kredlow and Otto (2015)	Y	N	Y	Y	Y	Y	N	Y	Y	Y	Y	N/A	N
Surís et al. (2013)	Y	Y	N	Y	Y	Y	N	Y	N	Y	Y	N	Y
Wood et al. (2015; study 2)	Y	Y	N	Y	Y	Y	Y	Y	N/A	N	Y	Y	Y
Wood et al. (2015; study 3)	Y	Y	N	Y	Y	Y	N	Y	N/A	N	Y	Y	Y

Y = desirable study characteristic present; N = desirable characteristic not present. (A) Is the study design (or paradigm) described? (B) Detailed inclusion and exclusion criteria provided? (C) Procedures for randomisation described? (D) Procedures for blinding (if appropriate, i.e. if outcome is experimenter rated) described? (E) Primary outcome(s) clearly specified? (F) Relevant demographics for subjects provided? (G) Sufficient experimental control (i.e. both a no retrieval + treatment and a retrieval + placebo group included)? (H) Groups comparable at baseline? (I) Inter-rater reliability achieved and evaluated where relevant? (J) Duration of retrieval trial provided (or reference made to duration from previous published studies)? (K) Treatment ‘dose’ provided? (L) Timing of drug administration relative to reconsolidation clearly described? (M) Where relevant, missing data (> 20%) dealt with appropriately?

### Retrieval procedures

Most studies used in vivo exposure to CSs (e.g. powder resembling crack cocaine; a live spider), other visual representations of the CS (e.g. video of cocaine use; a series of pictures of spiders) or both to reactivate memories. All trauma-related studies encouraged autobiographical recall of the traumatic incident(s) to reactivate trauma memory. Other studies also incorporated instructions to recall specific relevant autobiographical episodes evoked by the CSs (e.g. Das et al. 2015a, 2015b, 2018a; Hon et al. 2016; Pachas et al. 2015; Telch et al. 2017), and five studies included an obvious prediction error procedure at retrieval (Das et al. 2015a, b, 2018a; Hon et al. 2016; Soeter and Kindt 2015a). The latter involved some form of expectation violation (e.g. generating an expectation that the participant will experience the US, and then violating this expectation; Das et al. 2018b).

As outlined in Tables 1 and 2, most studies provided some details about the duration of the retrieval procedure. The modal duration in substance use studies was 5 min (used in six of the seven studies specifying retrieval duration); one study used a longer retrieval procedure (2 × 10 min; Saladin et al. 2013). Eight of the 10 phobia/trauma studies specified the duration of the retrieval procedures, which varied more than the substance use studies. All phobia studies used ≤ 2-min retrievals, with most studies clustered in the 5–15-s range. The three trauma-related studies that specified retrieval duration used 4, 10–20 and 30–75 min (the relevance of the length of the retrieval procedure is outlined in the discussion).

### Pharmacological and behavioural reconsolidation interference procedures

Pharmacological studies most commonly used propranolol [*k* = 2 substance use studies (Pachas et al. 2015; Saladin et al. 2013) and *k* = 2 phobia/trauma studies (Brunet et al. 2018; Soeter and Kindt 2015a)]. The reconsolidation-interfering effects of mifepristone (*k* = 2; Wood et al. 2015; studies 2 and 3) and sirolimus (rapamycin; *k* = 1; Surís et al. 2013) on threat memory and memantine (*k* = 1; Das et al. 2015a) and nitrous oxide (*k* = 1; Das et al. 2018a) on reward memory were also examined. In all cases, selection of these drugs by study authors was based on their putative downstream protein synthesis-inhibiting effects and, particularly, their tendency to interfere with the protein synthesis-dependent restabilisation phase of reconsolidation.

Among behavioural studies, retrieval-extinction was the most commonly tested procedure, either using ‘standard’ in vivo and/or picture-stimulus exposure in specific phobia (Bjorkstrand et al. 2017; Telch et al. 2017) and substance use (Germeroth et al. 2017; Xue et al. 2012) or virtual reality exposure for specific phobia (Maples-Keller et al. 2017; Shiban et al. 2015). The remaining behavioural studies examined post-retrieval counterconditioning (Das et al. 2015b) and cognitive reappraisal (Hon et al. 2016) in substance users (heavy alcohol drinkers) or prose interference (Kredlow and Otto 2015) in sub-clinical, trauma-exposed individuals.

### Study outcomes

Across all studies, 15 of the 18 ESs were positive (favouring retrieval-dependent reconsolidation interference). With the exception of Brunet et al. (2018), who reported high levels of attrition at 6-month follow-up, ESs are based on comparisons between the retrieval and control condition on the last assessed time point for the relevant outcome (the ES for the study of Brunet et al. (2018) was based on the penultimate follow-up). For the outcomes selected for the current meta-analysis, this ranged from 1 day (Shiban et al. 2015) to 12 months (Maples-Keller et al. 2017; Soeter and Kindt 2015a). We deemed this relatively stringent longest time point comparison to be appropriate given the claim for *permanent* memory modification following reconsolidation interference.

Among the phobia/trauma studies, other than outcomes from the BAT (phobia studies) and trauma symptom severity/trauma memory recall (trauma-related studies) used to calculate ESs, some of the reviewed publications reported additional outcomes showing significant retrieval-dependent benefits (Table 1). These included reduced skin conductance in response to fear-provoking stimuli (Maples-Keller et al. 2017), subjective fear/phobic symptoms (Soeter and Kindt 2015a) and neural activity in the amygdala (Bjorkstrand et al. 2016, 2017). Notably, reductions in subjective fear (of spiders) in the study of Soeter and Kindt (2015a) only emerged at long-term follow-up, suggesting a lagged benefit for some outcomes following reconsolidation interference treatments. Conversely, Maples-Keller et al. (2017) reported relatively *higher* physiological arousal (heart rate) at a 3-month follow-up in the retrieval + treatment (exposure) group, in the absence of, as well as during, exposure to feared cues. However, this was interpreted as a relative benefit to the retrieval group (i.e. high levels of fear were thought to attenuate physiological reactivity in the no retrieval group, although there were no between-groups differences in fear ratings at this time point).

In addition to craving, other statistically significant effects were also reported in a number of the substance use studies (Table 1). These included reductions in smoking (Germeroth et al. 2017), alcohol attentional bias (Das et al. 2015b), alcohol cue liking (Das et al. 2015b), fluency for positively valenced alcohol words (Hon et al. 2016) and cocaine and heroin cue-evoked blood pressure changes (Saladin et al. 2013; Xue et al. 2012).

### Control conditions

A control group that received treatment in the absence of putative reactivation (no retrieval + treatment) was considered the most appropriate comparison condition and was the most commonly employed. A number of pharmacological studies used a retrieval + no treatment (placebo) group (Brunet et al. 2018; Pachas et al. 2015; Saladin et al. 2013; Surís et al. 2013; Wood et al. 2015; study 3), as did Kredlow and Otto (2015), who compared negatively valenced interfering prose with a no prose condition.

### Effect size for symptoms of phobia and trauma

**Fig. 2:**
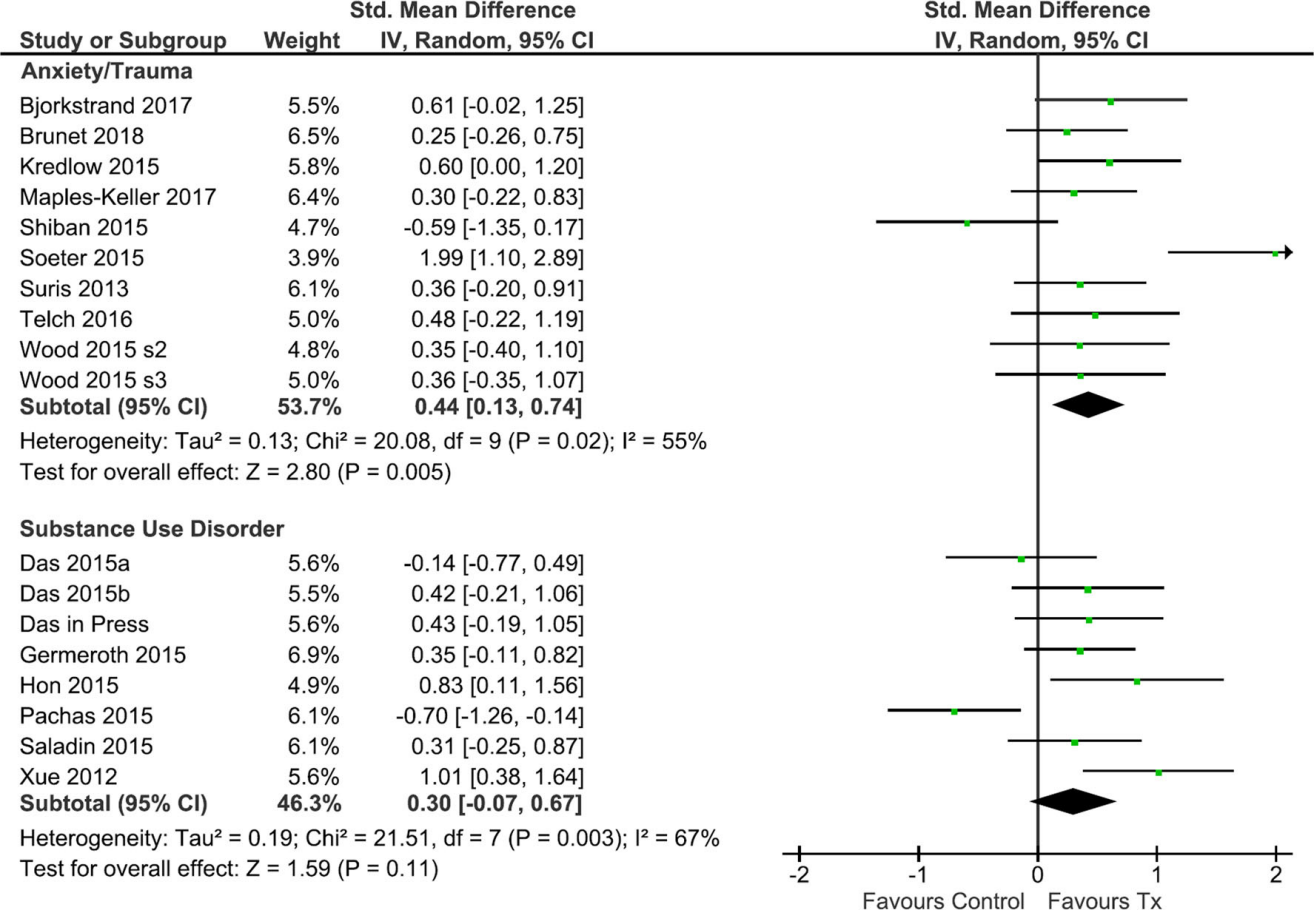
Forest plot of all included studies and a comparison of overall ES for phobia/trauma and substance use studies

The aggregate ES for phobia/trauma symptoms was medium (*k* = 10; *n* = 402; *g* = 0.44, 95% CI [0.13, 0.74], *p* = 0.005; Fig. 2) and showed moderate heterogeneity (*I*^2^ = 55%). It is clear from inspection of the forest plots, however, that the study of Soeter and Kindt (2015a) contributes disproportionately to the overall ES. A sensitivity analysis showed that exclusion of Soeter and Kindt (2015a) eliminated heterogeneity (*I*^2^ = 0%) but also reduced the ES (*g* = 0.33, 95% CI [0.12, 0.53]), although it remained significantly > 0 (*p* = 0.002).

**Fig. 3:**
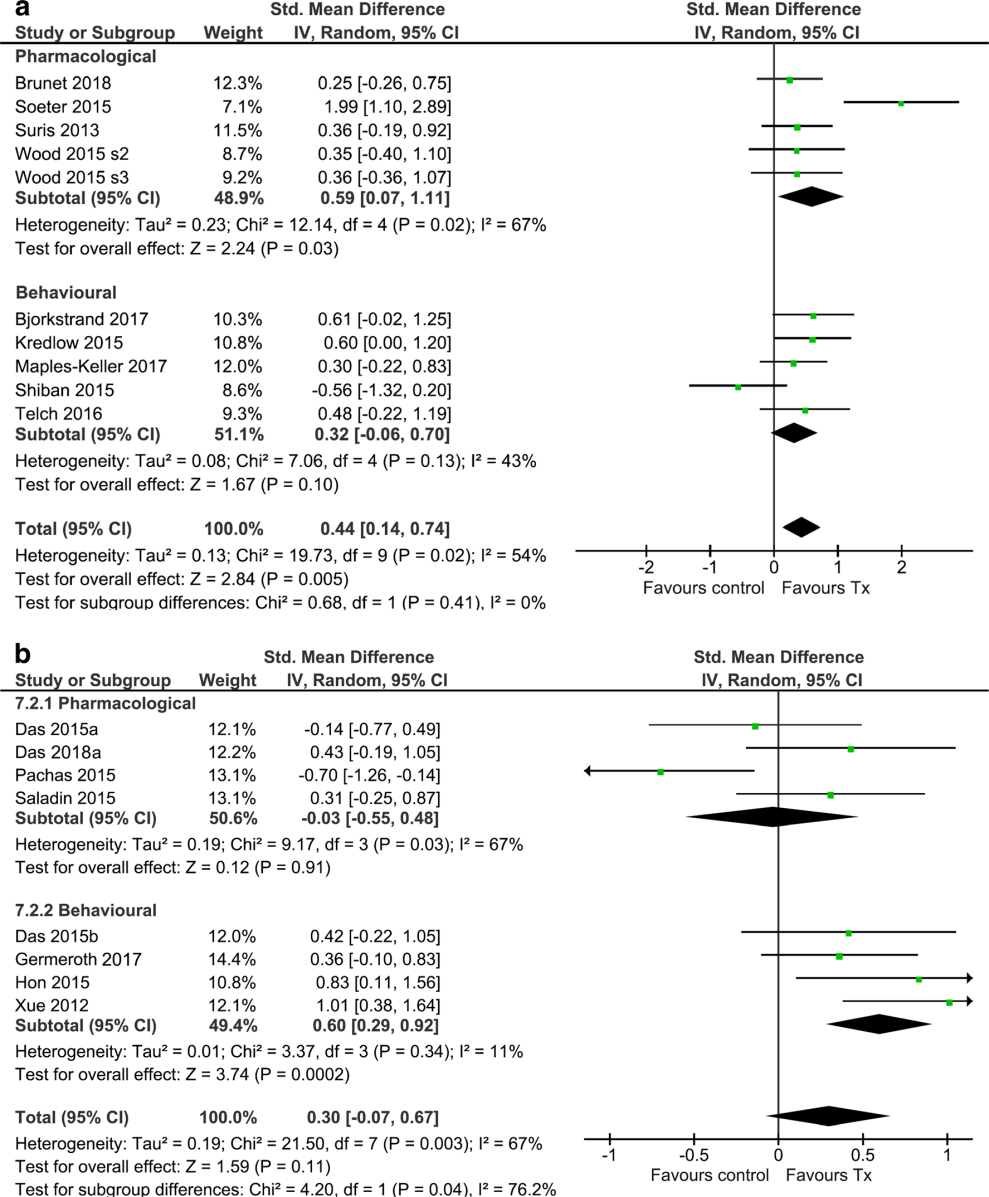
**a** Comparative forest plot for treatment type (behavioural vs. pharmacological) in studies of maladaptive threat memories (phobia/trauma). **b** Comparative forest plot for treatment type (behavioural vs. pharmacological) in studies of reward memories (substance use)

The aggregate ES for all pharmacological studies was medium (*k* = 5, *g* = 0.59, 95% CI [0.07, 1.11], *p* = 0.03), and heterogeneity was relatively high (*I*^2^ = 67%). When Soeter and Kindt (2015a) was retained in the analysis, the population ES estimate had poor precision, with the true effect lying in the range from very small to large-very large (Fig 3a). Exclusion of Soeter and Kindt (2015a) eliminated heterogeneity (*I*^2^ = 0%) but also reduced the ES, although it remained significant (*k* = 4, *g* = 0.32, 95% CI [0.01, 0.62], *p* = 0.04). A small, non-significant ES was found for behavioural studies of phobia/trauma (*k* = 5, *g* = 0.32, 95% CI [− 0.07, 0.70], *p* = 0.10), with a moderate degree of heterogeneity (*I*^2^ = 46%). Sub-group analysis showed that pharmacological and behavioural studies did not significantly differ, regardless of the inclusion (*χ*^2^(1) = 0.69, *p* = 0.40) or exclusion (*χ*^2^(1) = 0.00, *p* = 1.00) of Soeter and Kindt (2015a).

### Effect size for symptoms related to substance use

Across all substance use studies (Fig. 3b), the aggregate ES was small and non-significant (*k* = 8; *n* = 372; *g* = 0.30, 95% CI [− 0.07, 0.67], *p* = 0.11; Fig. 2), with relatively high levels of heterogeneity (*I*^2^ = 67%). Sensitivity analysis identified a single study (Pachas et al. 2015) that appeared to be especially influential. Its removal reduced heterogeneity to *I*^2^ = 24% and increased the aggregate ES to a significant, small-moderate magnitude (*k* = 7; *n* = 318; *g* = 0.44, 95% CI [0.18, 0.70], *p* < 0.001).

Sub-group analysis of substance use studies indicated that pharmacological studies (including Pachas et al. 2015) were associated with a negligible ES (*k* = 4; *n* = 184; *g* = − 0.03, 95% CI [− 0.55, 0.48], *p* = 0.91) and moderately high heterogeneity (*I*^2^ = 67%). The three pharmacological studies other than Pachas et al. (2015) had a small combined ES, which was non-significant (*g* = 0.21, 95% CI [− 0.14, 0.56], *p* = 0.23). In contrast, behavioural studies yielded a significant, medium ES (*k* = 4; *n* = 188; *g* = 0.60, 95% CI [0.29, 0.92], *p* < 0.001), with low heterogeneity (*I*^2^ = 11%). A moderator analysis including Pachas et al. (2015) suggested that the ESs of behavioural and pharmacological studies of substance use were significantly different (*χ*^2^(1) = 4.20, *p* = 0.04; Fig. 3), although removal of this statistically influential study brought the two effect sizes closer together such that moderation by treatment was no longer significant (*χ*^2^(1) = 2.63, *p* = 0.11).

### Moderation

Across *all* studies, meta-regression suggested that none of the specified moderators (age, proportion of male participants or methodological appraisal score) were significant predictors of ES (*t* values < 1.5, *p* values > 0.1).

### Publication bias

**Fig. 4:**
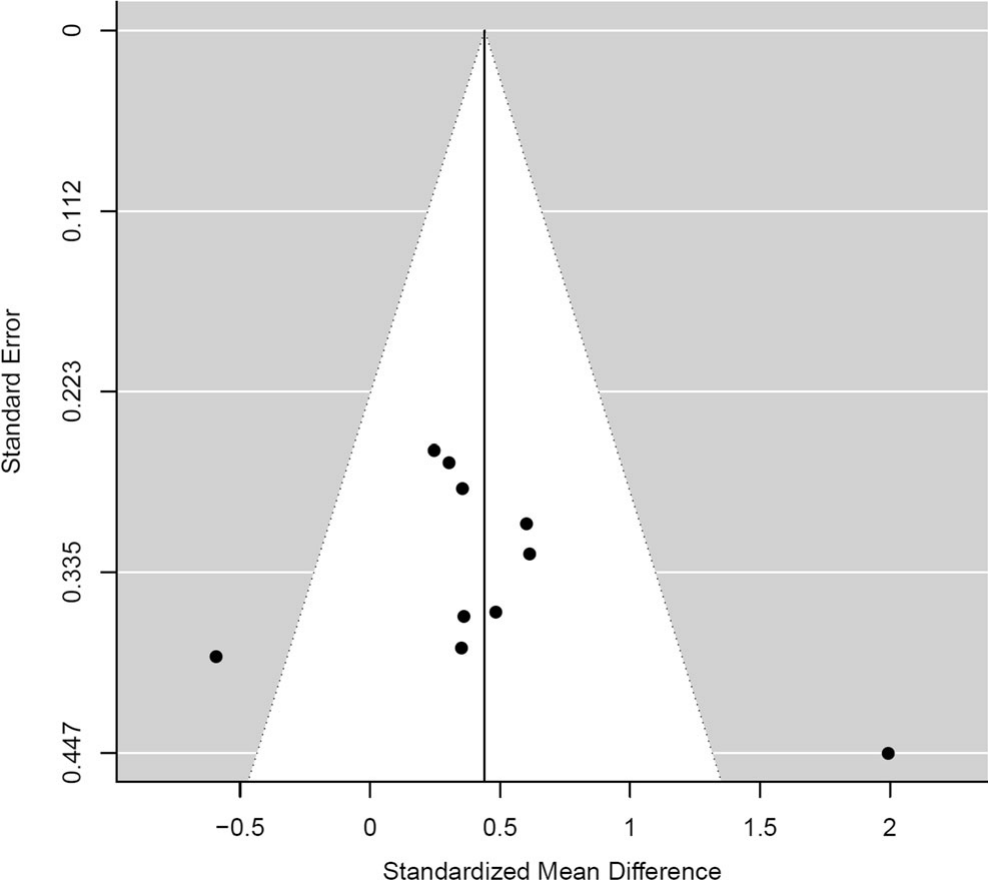
Funnel plot of ES against the standard error for studies of phobia/trauma

**Fig. 5:**
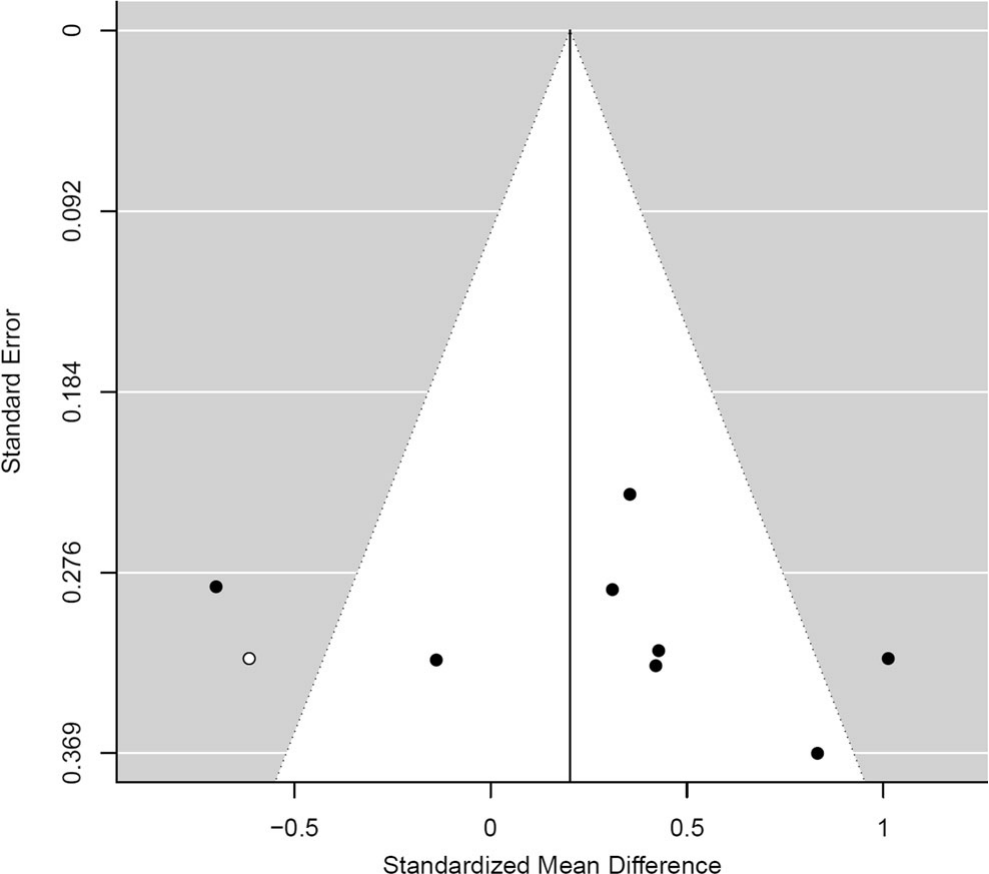
Funnel plot of ES against the standard error for studies of substance use

A funnel plot for the phobia/trauma studies did not indicate asymmetry (*t*(8) = 0.22, *p* = 0.831; Fig. 4). No adjustments to the effect of phobia/trauma studies was suggested by trim and fill (Duval and Tweedie 2000a, b). The funnel plot for studies of substance use similarly indicated a lack of asymmetry (*t*(6) = 0.956, *p* = 0.367; Fig. 5), with trim and fill, suggesting one study with a negative ES was absent. No adjustment to effect was observed following the exclusion of Pachas et al. (2015). Overall, these results suggest an absence of publication bias for phobia/trauma and substance use studies.

## Discussion

This meta-analysis provides a synthesis and critical evaluation of research on reconsolidation of naturalistic maladaptive memories using pharmacological and behavioural memory-weakening/interfering strategies in (sub) clinical samples. Extension of non-human and human experimental findings to clinically relevant populations is a relatively new area of translational research, with the oldest publication in this review dated 2012. As such, there are currently a small number of relevant studies, although findings across these were relatively consistent. In particular, 15 of 18 ESs were in the predicted direction (i.e. favouring a reconsolidation-modulation interpretation), but of the two broad disorder categories, only the population ES estimate for phobia/trauma (across behavioural and pharmacological studies) was significant. Moderator analysis by intervention type (pharmacological versus behavioural) indicated statistically larger effects in behavioural versus pharmacological studies in the case of substance use, while the opposite pattern was observed for phobia/trauma studies, with pharmacological treatments producing an almost twofold larger ES than behavioural treatments (although this comparison was not statistically significant). However, these general findings need to be considered in the context of the finding that the ES for each ‘disorder type’ was substantially influenced by a single study that inflated heterogeneity and skewed the results towards either a larger (phobia/trauma) or smaller (substance use) ES.

### General overview of studies

Across all substance use studies, the overall ES was small and non-significant, although removal of Pachas et al. (2015) reduced heterogeneity and increased the aggregate ES estimate, rendering it significant. Phobia/trauma studies had a significant, medium ES when all studies were considered, and a significant, small-medium ES when Soeter and Kindt (2015a) was excluded. Overall, these findings support the idea that, despite their chronological age and strength relative to experimentally acquired memories, naturalistic maladaptive memories are capable of being destabilised and subsequently weakened/overwritten using reconsolidation-modulating strategies. As such, the putative boundary conditions (memory remoteness and strength) that appear to limit the ‘destabilisation potential’ of experimentally trained memories in non-human animals (Milekic and Alberini 2002; Suzuki et al. 2004; Vousden and Milton 2017) do not necessarily preclude destabilisation of naturally acquired memories in humans, although they might constrain the degree of destabilisation and, hence, the magnitude of intervention effects. This provisional conclusion is promising for the development of such strategies as therapeutic interventions for threat-related and substance use disorders.

However, ESs varied considerably across substance use and phobia/trauma studies. This might have reflected the substantial variation in retrieval procedures (e.g. retrieval duration; timing of treatment relative to retrieval, number of treatment sessions), the nature of reconsolidation interference strategies (i.e. the use of different drug classes and behavioural interventions) and participant characteristics. As such, there continues to be uncertainty about optimal retrieval parameters and/or retrieval-dependent interference strategies required to interfere effectively with naturalistic maladaptive memories. Such memories are likely to constitute highly distributed traces, involve multiple memory systems (semantic, autobiographical-episodic, priming/implicit; varying in affective valence) and vary substantially in strength and remoteness from one individual to another. This is clearly very different to the situation in studies of laboratory-trained memories, in which learning across participants is uniform and usually involves a limited set of stimuli from well-defined categories (e.g. sets of simple sensory stimuli as CSs) within a single context. Moreover, effective retrieval (reactivating) cues in laboratory studies are simply those that were used during training, whereas the nature of *suitable* (i.e. optimal) retrieval cues for naturalistic memories is unclear. Given the uncertainty regarding suitable retrieval conditions for naturalistic memories, as well as the likelihood that such memories are more strongly entrained (over a long period) than experimentally acquired memories, it might be expected that ESs would differ between experimental and naturalist memories. It is therefore instructive to compare our ES estimates with those obtained in previous meta-analyses of reconsolidation studies of experimentally acquired memories in humans. The results reported in two relevant meta-analyses on emotional (threat-related) memories are therefore considered (Kredlow et al. 2016; Lonergan et al. 2013) in relation to phobia/trauma studies.

### Phobia/trauma studies

Kredlow et al. (2016) examined changes in conditioned fear in studies employing the prototypical behavioural reconsolidation procedure, retrieval-extinction (Monfils et al. 2009). Their reported aggregate ES, relating to tests of return of fear, is not dissimilar to that for behavioural strategies (also most commonly, retrieval-extinction) employed in the phobia/trauma studies reviewed here. By contrast, the aggregate ES reported here for phobia/trauma studies using pharmacological treatments (propranolol, mifepristone and sirolimus; *g* = 0.71) was somewhat larger than that reported in a meta-analysis of studies of the effects of propranolol on memory for negatively valenced words and cue-induced fear responding (*g* = 0.56; Lonergan et al. 2013). Heterogeneity of ESs was broadly similar between the currently reviewed behavioural studies and those in Kredlow et al. (2016) and between the current pharmacological studies and those in Lonergan et al. (2013). These broad similarities underscore the potential for applying research findings on experimentally acquired memories to naturalistic memories in clinical disorders. However, they also highlight the need for further research to identify common sources of heterogeneity in ESs in reconsolidation research.

As noted above, a single influential study (Soeter and Kindt 2015a) contributed disproportionately to the medium ES of pharmacological studies summarised here. Removal of this study substantially reduced the ES. However, given that this study produced the most pronounced, prolonged (1 year) and generalised (across behavioural and subjective-evaluative indices of fear memory) effects, it is worth considering the study features that might have contributed to these particularly large and durable effects. It is noteworthy, for example, that Soeter and Kindt (2015a) used post-retrieval propranolol as a pharmacological interference strategy, and it was also the only pharmacological study on specific phobia rather than PTSD. The use of propranolol by these authors was based on their multiple previous demonstrations of reconsolidation impairing effects of propranolol on experimentally acquired memories (conditioned fear; Kindt et al. 2009; Sevenster et al. 2012, 2013, 2014; Soeter and Kindt 2010, 2011, 2012a, b, 2015a; but see Bos et al. 2014; Schroyens et al. 2017). The use of *post-retrieval* propranolol may be particularly relevant to the large effects seen in this study. While specific NMDAR sub-units have repeatedly been shown to be involved in destabilisation (see below), the role of β-adrenergic signalling in this first phase of reconsolidation has been unclear. However, there is some recent indirect support for the idea that adrenoceptors might also be involved in destabilisation and that their premature blockade might therefore constrain reconsolidation interference effects (Lim et al. 2018). If this is true, post-retrieval, rather than pre-retrieval propranolol administration is likely the optimal strategy for reconsolidation interference-based therapies.

While fear conditioning reconsolidation studies that have used propranolol have demonstrated that relatively brief (< 10-s) retrievals involving unreinforced presentation of a CS appear sufficient to reactivate conditioned fear memories, Soeter and Kindt (2015a) used a longer retrieval duration (2 min), as is apparently required for reactivation of chronologically remote memories (Suzuki et al. 2004). This was substantially longer than the retrievals used in the other (behavioural) phobia studies reviewed here (Bjorkstrand et al. 2016; Maples-Keller et al. 2017; Shiban et al. 2015; Telch et al. 2017). Soeter and Kindt’s (2015a) retrieval procedure also involved a prediction error (spider phobic participants expected that they would touch a spider during retrieval, but in fact, this did not occur). Finally, the retrieval procedure involved in vivo exposure to a spider (cf. Bjorkstrand et al. 2016; Maples-Keller et al. 2017; Shiban et al. 2015), which, as a biologically ‘prepared’ stimulus, might be considered to possess qualities of a US. Some researchers (e.g. Liu et al. 2014) have suggested that the use of USs at retrieval results in a more generalised destabilisation of relevant associations (between all CSs and the US). This might also explain the more generalised effects on fear responding observed in Soeter and Kindt (2015a). A close replication is now required to establish that this combination of factors (i.e. use of post-retrieval propranolol, medium duration retrieval and/or use of cues with US properties and/or incorporating a relevant prediction error at retrieval) reliably produces large and durable reconsolidation effects on naturalistic fear memories. Thereafter, studies might seek to determine if this combination is *required*.

The basic behavioural pharmacology of other neurotransmitter/neuromodulator systems in reconsolidation is less developed relative to the noradrenergic system. For example, despite the established role of glucocorticoid stress system in memory, disruption of which is implicated in psychological disorders (de Quervain et al. 2016), few experimental studies have been conducted on the role of this system in reconsolidation in humans. Moreover, endogenous and exogenous corticosteroids have a variety of distinct and opposing effects on memory (e.g. impairment of retrieval versus enhancement of (re)consolidation), depending upon, for example, the timing of the glucocorticoid surge relative to retrieval, the number (or duration) of CS exposure at retrieval (e.g. Cai et al. 2006) and background levels of arousal (see Meir Drexler and Wolf 2017). These multiple determinants of glucocorticoid effects might explain the conflicting results reported in existing human and non-human animal studies of glucocorticoid modulation of reconsolidation (de Quervain et al. 2016). It is unclear whether these considerations were relevant to the two studies of Wood et al. (2015; studies 2 and 3), both of which showed no statistical effect of mifepristone, a glucocorticoid receptor antagonist (although effects were in the predicted direction). In Wood et al. (2015; study 3), the authors additionally attempted to augment the impairing effects of mifepristone through pre-treatment with the NMDAR (glycine site) partial agonist, d-cycloserine. This strategy might be particularly relevant when long-term allostatic processes (Espejo et al. 2016) result in an enduring downregulation of NMDAR (NR2B) sub-units, which are required for memory destabilisation (Ben Mamou et al. 2006; Wang et al. 2009). As is evident from Figs. 2 and 3a, DCS did not appear to affect mifepristone’s ability to interfere with reconsolidation of trauma memory.

The limited effects of mifepristone might be attributable to specific procedural factors in the two studies described in Wood et al. (2015). For example, the use of individualised scripts likely introduced variability in retrieval duration across participants. In addition, apparently prolonged ‘script preparation’ procedures at retrieval (writing about two traumatic experiences from the same or different events and recalling subjective, visceral and muscular reactions associated with these experiences) might have engaged extinction rather than reconsolidation processes. Alternatively, *relatively* prolonged (intermediate) retrieval durations can also engage a so-called ‘limbo state’ (Merlo et al. 2014), in which neither extinction nor reconsolidation is engaged. These limitations in the extant research on drugs that downregulate glucocorticoid receptor activity do not allow firm conclusions to be drawn about their application as reconsolidation-interfering treatments at this stage.

Unlike the role of the noradrenergic and glucocorticoid systems, little is known about the effects of manipulating the mTOR pathway on any aspect of memory functioning in humans. While rapamycin blocks fear-related memory reconsolidation in rodents (e.g. Blundell et al. 2008), this capacity has not yet been established in experimental studies of emotional learning and memory in humans. This makes it difficult to interpret the limited efficacy of rapamycin reported in Surís et al. (2013). In addition to the long retrieval duration (up to 75 min) used in that study, the pharmacokinetic profile of rapamycin (i.e. its central bioavailability after a single 15 mg oral dose) relative to the timing of destabilisation (assuming this actually occurred) may not have been optimal.

In contrast to the ES estimate from pharmacological studies of phobia/trauma, the population ES from studies of post-retrieval behavioural strategies was small (approximately half that obtained from pharmacological studies) and not significantly different from 0. Despite four of the five ESs favouring the retrieval + treatment group, the effect was skewed towards a smaller value by the study of Shiban et al. (2015). The ES in that study was based on a spontaneous recovery test performed 1 day after retrieval-extinction. If there is a ‘sleeper effect’ on BAT performance, as found for declarative aspects of fear by Soeter and Kindt (2015a); note these authors tested behavioural approach for the first time 11 days after treatment with propranolol), retrieval-dependent effects might not have been evident after such a short interval. However, in contrast to Soeter and Kindt (2015a), Shiban et al. (2015) reported no evidence for stronger effects of retrieval-extinction on declarative fear after a long follow-up period (6 months). In addition to differences in the retrieval procedures between these two studies of spider phobics, (non-significantly) higher baseline levels of fear, heart rate and skin conductance, as well as lower baseline approach behaviour in the retrieval-extinction group (relative to the control group), might have contributed to limited extinction in the retrieval-extinction group in the study of Shiban et al. (2015).

One notable finding among the behavioural phobia/trauma studies was that described by Telch et al. (2017), who reported a relatively immediate reduction in fear responding (expectancy and peak fear) in the retrieval-followed-by-treatment (extinction) group relative to the treatment-followed-by-retrieval control group. These authors consider a number of explanations for this unexpected early effect (e.g. the occurrence of prediction error or increased noradrenergic activity resulting from the retrieval trial). However, it should also be noted that the interval between retrieval and the first extinction trial was especially long in this study (30 min). While the use of a delay between retrieval and the interference strategy is a common feature of behavioural reconsolidation studies (although the interval is usually only 10 min), the rationale for employing such a delay is unclear and may simply be a carryover from the procedure used in the first studies of retrieval-extinction in rodents and humans (Monfils et al. 2009; Schiller et al. 2010). Indeed, without employing *high cognitive load tasks* between the end of retrieval and the start of the interference task (e.g. Das et al., 2015b, 2018b; Hon et al. 2016), there is the potential for ongoing cognitive engagement/rehearsal following exposure to the reminder cue, possibly initiating extinction. This might therefore be an alternative explanation for the apparent early retrieval-dependent enhancement of extinction reported by Telch et al. (2017).

### Substance use studies

Despite the small number of studies of laboratory-based reward-memory reconsolidation in humans (e.g. Xue et al. 2017; Zhao et al. 2011), there is substantial evidence for reconsolidation modulation in rodent models of maladaptive reward/addiction. Meta-analytic findings on appetitive-reward memory in non-human animals suggest that the ES associated with reconsolidation interference using NMDAR antagonism is substantially larger than that for β-adrenergic antagonists (Das et al. 2013). This might explain the relatively modest and short-lived effects of propranolol reported by Saladin et al. (2013). Alternatively, the retrieval procedure in Saladin et al. (2013) was relatively protracted (2 × 10 min of in vivo and video cue exposure to cocaine cues), which might have limited plasticity through activation of extinction or generating a limbo state. Another study that used propranolol (Pachas et al. 2015) showed a *negative* ES (the propranolol group showing *higher* levels of craving relative to retrieval + no treatment). It should be noted, however, that the latter study used a script preparation protocol inspired by the same studies that informed the retrieval procedures of Wood et al. (2015). Again, while the duration of the retrieval procedure was not stated in Pachas et al. (2015), it is likely that it was also prolonged (and likely to vary between participants). As such, the potential for extinction/limbo state processes is again relevant. In addition, since craving was *higher* in the retrieval + treatment group, it is possible that (inadvertent) extinction consolidation was *impaired* by propranolol relative to the placebo group (Cahill et al. 2000).

Excluding Pachas et al. (2015), the remaining three pharmacological studies (Das et al. 2015b, 2018a; Saladin et al. 2013) showed no evidence of a combined effect consistent with reconsolidation interference. Based on the larger effects of NMDAR versus β-adrenergic antagonists on reward memory reconsolidation (Das et al. 2013), Das et al. (2015a) examined the NMDAR antagonists, memantine, but found no evidence for an effect consistent with reconsolidation blockade. It is unclear whether the typical therapeutic dose (10 mg) and route of administration (oral) of memantine is suitable for blocking reconsolidation in humans. Indeed, memantine has slow absorption kinetics and relatively low selectivity for the most abundant central NR2A NMDAR sub-unit (Ogden and Traynelis 2011), which is involved in restabilisation (Milton 2013), giving rise to uncertainty about whether suitable reductions in NMDAR activity were actually achieved in the post-retrieval period. The other putative NMDAR antagonist used in this group of studies, nitrous oxide (Das et al. 2018a), also did not show a reconsolidation blocking effect based on the planned statistical analysis. However, when the data was reanalysed to take account of whether participants in the retrieval groups experienced a prediction error at retrieval, significant retrieval-dependent effects of nitrous oxide were evident. Since the latter findings were not based on a pre-specified statistical analysis plan, the ES from this study used in the current meta-analysis, was based on the non-significant findings.

In contrast to pharmacological strategies, behavioural methods for interfering with reconsolidation showed more promise in the case of substance use. Indeed, the ES associated with behavioural studies was significantly larger than that of pharmacological studies, although this statistical finding needs to be treated with caution, given the small number of studies and lack of precision in the population ES estimate of pharmacological studies. Of the four behavioural substance use studies, two employed post-retrieval cue exposure (retrieval-extinction; Germeroth et al. 2017; Xue et al. 2012): one counterconditioning (Das et al. 2015b) and one cognitive reappraisal (Hon et al. 2016). All showed positive ESs on measures of craving, and the overall ES was moderate-large, with minimal heterogeneity. It is noteworthy that while the nature of the reactivating cues varied between studies (e.g. drug use video, in vivo drug cues, drug pictures or a combination of these), all used the same retrieval duration (5 min), along with a 10-min interval between termination of retrieval and start of the behavioural strategy. As such, these retrieval parameters can be recommended for future substance use reconsolidation studies, at least until optimal parameters are firmly established through studies that parametrically vary retrieval parameters.

In addition, it is noteworthy that all of the substance use behavioural interference studies reported significant effects on more than one outcome. Indeed, Das et al. (2015b) reported results consistent with a comprehensive rewriting of affective, attentional and cognitive aspects of alcohol-related memories in heavy drinkers. However, the follow-up period for this study, along with Hon et al. (2016), was relatively short (1 week), whereas the other two studies tested participants at 1 month (Germeroth et al. 2017) and 6 months (Xue et al. 2012). Three of the studies examined changes in substance use behaviour (Das et al. 2015a; Germeroth et al. 2017; Hon et al. 2016), but only one of these showed significant changes in drug (cigarette) use (Germeroth et al. 2017). The latter study, along with the study of Xue et al. (2012), used a two-session treatment protocol (two retrieval + treatment sessions). As such, despite low levels of heterogeneity in ESs of these behavioural studies, there was considerable methodological variation. It remains to be determined whether counterconditioning and cognitive reappraisal result in sustained effects on craving [and/or in attentional bias and effective responding to alcohol (Das et al. 2015b) and semantic memory for alcohol (Hon et al. 2016)] and whether behavioural effects might also emerge after a longer delay.

### Limitations

The effects reported here are based on a relatively small number of studies in each category of disorder (these were further reduced in the treatment-type moderator analyses). In addition, the studies themselves generally had small sample sizes. Moderation was only examined for a small number of covariates in the current analysis. However, assuming detailed methodological reporting in future studies, meta-analysis/regression based on a larger number of studies with greater variability in retrieval variables might prove to be a particularly effective way of establishing the role of retrieval parameters (e.g. retrieval duration; use of prediction error at retrieval) in successful memory reactivation. The alternative (and preferable) approach would involve a parametric variation of these retrieval parameters within individual studies, although this approach would require very large sample sizes due to the number of potential factors and levels of key retrieval variables that would be manipulated.

In contrast to the array of outcomes reported in the reviewed studies, our analysis focused on a narrow set of pre-determined outcomes, primarily trauma symptoms in studies of trauma-exposed individuals, behavioural approach or subjective fear (based on BAT performance) in phobia studies and craving in the case of substance use studies. We opted to base our ES calculations on these outcomes rather than those reported as statistically significant in publications. However, it is possible that quite different results would be achieved if only the significant results reported by study authors (either singly or as composites of multiple significant outcomes) were used to determine ESs. This is not necessarily a limitation, as our intention was to reduce the potential for bias in cases where multiple outcomes were reported, but none was pre-specified as primary.

### Recommendations for future research

The strength of the reviewed studies was their tendency to recruit participants in line with the relative gender prevalence of disorders in question. However, it should be noted that recent evidence suggests that men and women may be differentially susceptible to some reconsolidation-interfering treatments. In particular, Meir Drexler et al. (2016) showed that whereas men showed retrieval-dependent weakening following hydrocortisone, this effect was absent in women. It is currently unclear whether this finding is specific to glucocorticoid modulation of reconsolidation, rather than reflecting a general insensitivity to reconsolidation interference in women. Indeed, the latter seems highly unlikely, given the very large effects seen with propranolol in Soeter and Kindt (2015a), whose sample consisted almost exclusively of women (91%). No individual study that we are aware of has yet examined gender moderation in (sub) clinical populations, although this seems a particularly important factor to consider if reconsolidation interference is to be used clinically. It should be noted provisionally that our moderation analysis did not suggest an effect of gender.

Among the pharmacological studies, there was no common use of a single reconsolidation-interfering drug. Although propranolol was most commonly studied, this amounted to only two studies in the substance use category and two studies in the phobia/trauma grouping. As such, it remains unclear whether one drug class category might be more effective in preventing restabilisation than others. Despite strong evidence from studies with non-human animals, only two of the reviewed studies examined an NMDAR antagonist. Given the central role of glutamatergic neurotransmission in learning and memory, further research on the effects of NMDAR antagonist effects on reconsolidation in humans seems to be a special priority. On the other hand, clinical studies should be preceded by more basic psychopharmacological studies in order to determine the importance of drug timing (relative to retrieval) and route of administration. This is particularly important given the potential for iatrogenic effects of NMDAR antagonists (i.e. the potential for paradoxical strengthening of maladaptive memories in some contexts (Honsberger et al. 2015)).

Despite its reported importance in memory destabilisation, a minority of the reviewed studies examined the role of prediction error (cf. Das et al. 2015a, b, 2018a; Hon et al. 2016; Soeter and Kindt 2015a). As noted previously, if there is a requirement for an optimal learning signal at retrieval, those studies showing beneficial effects of retrieval + treatment in the absence of prediction error might, in fact, represent the lower bound of efficacy that could be achieved during reconsolidation modulation. As such, tailoring retrieval procedures to maximise PE may bolster the likelihood that reconsolidation can be leveraged for clinical benefit.

Overall, our findings suggest that reconsolidation interference is worth pursuing as a clinical strategy. However, before proceeding with costly and labour-intensive clinical trials, the multiple sources of uncertainty regarding determinants of efficacy of this approach should be more thoroughly investigated through basic experimental human and animal research to ensure that studies with clinical populations are optimised and, therefore, as informative as possible.
